# Multi-Source Confidence Assessment-Based Adaptive Calibration for Deep-Sea Manned Submersible Integrated Navigation

**DOI:** 10.3390/s26041359

**Published:** 2026-02-20

**Authors:** Yixu Liu, Wentao Fu, Shengya Zhao, Yongfu Sun

**Affiliations:** 1 National Deep Sea Center, Qingdao 266237, China; lyx@ndsc.org.cn (Y.L.); zsy@ndsc.org.cn (S.Z.); sunyongfu@ndsc.org.cn (Y.S.); 2 College of Intelligent Systems Science and Engineering, Harbin Engineering University, Harbin 150006, China

**Keywords:** submersible, assessment of confidence, navigation and positioning, USBL

## Abstract

To address the insufficient reliability of manned submersible navigation systems in complex deep-sea environments, this paper proposes an adaptive fusion navigation method based on multi-dimensional confidence assessment. This study proposes a method establishing a four-dimensional evaluation framework for the USBL (Ultra-Short Baseline) positioning system. The framework encompasses signal quality, geometric precision, environmental attenuation, and data stability. It enables the quantitative, real-time assessment of system reliability. Consequently, it facilitates an adaptive weight adjustment mechanism. Experimental results demonstrate that under harsh conditions featuring jump point anomalies and data loss, the proposed algorithm achieves an average position error of 1.15 m. This represents a 53.1% improvement over conventional methods, with the enhancement reaching 58.9% in scenarios specifically affected by jump points. The proposed method study effectively enhances the navigation reliability of manned submersibles in complex underwater acoustic environments, thereby demonstrating significant engineering application value.

## 1. Introduction

The deep-sea represents a new strategic frontier for nations, harboring critical resources including minerals, biological organisms, and genetic materials. The exploration and development of such deep-sea resources require the mastery of advanced deep-sea detection technologies [1]. A variety of submersibles—including manned, unmanned, and tethered platforms—are progressively enabling access to deep-sea environments [2]. Among these, deep-sea manned submersibles have achieved the capability to operate at full ocean depths across the world’s major oceans, making them indispensable platforms for conducting complex tasks such as detailed seafloor observation, geological sampling, pipeline inspection, and shipwreck archeology [3,4]. The success of such missions, which heavily rely on real-time human judgment onboard, is critically dependent on precise and reliable navigation and positioning systems [5,6]. Accurate navigation not only ensures the spatial accuracy of scientific data, but also serves as a lifeline for the safe operation of submersibles in complex seafloor environments.

A single navigation system struggles to support high-precision positioning requirements independently throughout the entire lifecycle. Although the SINS (Strapdown Inertial Navigation System) operates fully autonomously, errors accumulate in it over time [7,8]. Although acoustic positioning systems such as USBL provide absolute position references, they remain susceptible to complex acoustic environments [9]. Favored for its lightweight equipment and lower cost, USBL has become the mainstream method for acquiring underwater positioning information. However, practical applications often involve issues such as data packet loss, delays and intermittent signals, which severely compromise navigation continuity and reliability. Extensive research on acoustic error correction for underwater positioning has been conducted by numerous scholars worldwide, effectively enhancing positioning accuracy [10,11]. Consequently, the use of multi-sensor integrated navigation technology to achieve the complementary advantages of information fusion has become essential for the construction of high-precision, reliable and robust underwater navigation systems [12]. While SINS/DVL (Doppler Velocity Log)/USBL combinations are theoretically optimal [13,14,15,16], their practical performance critically depends on the reliability of sensor measurements, particularly the highly variable USBL data.

In existing research, Zheng systematically analyzed USBL positioning errors and their application in submersible docking, establishing the relationship between random errors and measurement inaccuracies, and proposed methods to enhance accuracy [17]. Building on this foundation, M. Morgado investigated the error propagation processes of both USBL and SINS. By employing an extended Kalman filter with feedback correction, they compensated for random drift errors, thereby improving the navigation and positioning accuracy of the vehicle to some extent [18]. Zhang derived a centralized filtering algorithm for INS/USBL/DVL integration, though it suffered from issues such as model complexity and poor fault tolerance [19]. Liu established a tightly coupled SINS/USBL model, which effectively estimated sound velocity errors [20]. Meanwhile, Wang introduced Student’s t-distribution and proposed a robust adaptive filtering method to handle heavy-tailed noise, thereby enhancing both the accuracy and robustness of the system [21]. In order to address anomalies in USBL data, research has shifted towards robust and adaptive filtering algorithms, such as robust Kalman filtering and its optimized variants. While these methods enhance system robustness by intelligently handling outliers, their performance is limited by the requirement for precise calibration of time-varying noise parameters. Furthermore, their high computational complexity compromises their ability to operate in real time [22]. Concurrently, research has focused on correcting USBL errors at the source, primarily through the online estimation of sound velocity errors and differential USBL techniques. While these approaches substantially improve the accuracy of the raw data, differential methods usually require seabed reference stations to be used, which increases the complexity and cost of system deployment [23].

In summary, although existing research has made certain progress in integrated navigation methods, the following limitations persist: on the one hand, the assessment of USBL positioning quality typically relies solely on the signal-to-noise ratio (SNR), failing to comprehensively reflect its reliability under the influence of factors such as sound speed profile errors and ray bending. On the other hand, manned submersibles in practical operations still depend on manual judgment for whether to incorporate USBL calibration, resulting in delayed responses and strong subjectivity. To address these shortcomings, this paper proposes an adaptive calibration method based on multi-dimensional confidence assessment. By constructing a comprehensive confidence index to dynamically adjust the observation weights in the Kalman filter and designing a three-level decision-making mechanism to replace manual intervention, the method achieves intelligent fusion of USBL data and autonomous calibration of the navigation system.

## 2. Theory

### 2.1. SINS/USBL/DVL Integrated Navigation Filtering System

The SINS/USBL/DVL integrated navigation system employs a centralized filtering framework, aimed at mitigating the accumulated errors of the SINS through information fusion. The system utilizes the absolute position from the USBL and the relative velocity measurements from the DVL as observations to correct the navigation errors of the SINS. Firstly, the state equation describing the error propagation of the SINS is established [21]:

(1) $$\dot{X}=FX+GW$$

where the state vector ***X*** includes the state observations for SINS, USBL, and DVL:

(2) $$X={{\left[\begin{array}{ccc} X_{\mathrm{SINS}} & X_{\mathrm{USBL}} & X_{\mathrm{DVL}} \end{array}\right]}_{1\times 22}}^{T}$$

where the 15-dimensional state vector for SINS is defined as follows:

(3) $$X_{\mathrm{SINS}}={\left[\begin{array}{ccccc} \delta P^{T} & \delta V^{T} & \varphi^{T} & \varepsilon^{T} & \nabla^{T} \end{array}\right]}^{T}$$

where *δ**P*** denotes the position errors in three directions, *δ**V*** represents the velocity errors in three directions, φ indicates the attitude error angles in three directions, ***ε*** symbolizes the random drifts of gyroscopes along the *x*, *y*, and z axes of the carrier coordinate system, and ∇ refers to the biases of accelerometers.

The 6-dimensional state vector for USBL is defined as

(4) $$X_{\mathrm{USBL}}={\left[\begin{array}{cccccc} \delta \theta_{x} & \delta \theta_{x} & \delta \theta_{x} & \delta \alpha_{a} & \delta \beta_{a} & \delta D \end{array}\right]}^{T}$$

where $\delta \theta =\left[\begin{array}{ccc} \delta \theta_{x} & \delta \theta_{y} & \delta \theta_{z} \end{array}\right]$ represents the installation error angles in three directions, $\delta \alpha_{a}$ and $\delta \beta_{a}$ denote angle measurement errors, and *δD* indicates the range measurement error. Note that since the installation error angles, angle measurement errors, and USBL range accuracy errors are all small quantities, they can be treated as constants.

The state vector for the DVL is defined as

(5) $$X_{\mathrm{DVL}}=\left[\delta K\right]$$

where *δK* represents the scale factor error of the DVL.

In Equation (1), ***F*** is the system matrix, while ***G*** and ***W*** are the noise matrices related to the filter.

(6) $$F=\left[\begin{array}{ccc} F_{\mathrm{SINS}} & 0_{15\times 6} & 0_{15\times 1}\\ 0_{6\times 15} & F_{\mathrm{USBL}} & 0_{6\times 1}\\ 0_{1\times 15} & 0_{1\times 6} & F_{\mathrm{DVL}} \end{array}\right]$$

In addition to the state Equation (1), the observation equation for the SINS/USBL/DVL integration is

(7) Z=HX+V

where ***V*** is the filter noise-related term.

(8) $$Z=\left[\begin{array}{c} Z_{\mathrm{SINS}/\mathrm{USBL}}\\ Z_{\mathrm{SINS}/\mathrm{DVL}} \end{array}\right]$$

(9) $$H=\left[\begin{array}{c} H_{\mathrm{SINS}/\mathrm{USBL}}\\ H_{\mathrm{SINS}/\mathrm{DVL}} \end{array}\right]$$

where ***Z***_SINS/USBL_ and ***H***_SINS/USBL_ are the joint measurement equation and observation matrix for SINS and USBL, respectively. Correspondingly, ***Z***_SINS/DVL_ and ***H***_SINS/DVL_ are the joint measurement equation and observation matrix for SINS and DVL. Under ideal conditions where the model is accurate and the noise statistical characteristics remain stable, this framework can achieve optimal estimation of navigation errors. However, the core challenge in engineering practice lies in the complex deep-sea environment, which causes drastic changes in USBL measurement uncertainty, making it difficult to predefine its noise statistical characteristics accurately. As a result, the performance of fixed-parameter filters degrades significantly under such conditions.

### 2.2. Analysis of USBL Measurement Uncertainty and Its Impact on Integrated Navigation

Manned submersibles can obtain absolute position information through USBL systems. Among these, USBL has become the mainstream positioning method for manned submersibles supported by mother ships due to its advantages of high device integration, no need for seafloor deployment, operational flexibility, and low cost. The surface mother ship transmits the position information calculated by USBL to the submersible via acoustic communication technology at specific intervals, providing crucial external position calibration for the SINS/DVL integrated navigation system, thereby effectively suppressing the error accumulation of SINS. However, “convenience” and “accuracy” are difficult to achieve simultaneously in the USBL system. Its inherent working principle—calculating the target’s direction and distance by measuring the phase difference of acoustic wave arrival at a single baseline array—determines its relatively low positioning accuracy and high susceptibility to interference from complex underwater acoustic environments [17].

(10) $$\frac{\sqrt{\Delta {\bar{x}}^{2}}}{L}={\left[{\left(\frac{\Delta \bar{c}}{c}\right)}^{2}+{\left(\frac{\Delta \bar{t}}{t}\right)}^{2}+{\left(\frac{\Delta {\bar{\varphi }}_{x}}{\varphi }\right)}^{2}\right]}^{\frac{1}{2}}\cos\theta_{x}$$

where Δ*x* is the positioning error along the horizontal X-axis of the system, *L* is the slant range, *c* is the speed of sound, *t* is the propagation time, $\varphi_{x}$ is the phase difference of the received signal between adjacent array elements along the X-axis, and $\theta_{x}$ is the angle between the target slant range and the X-axis. The equation demonstrates that the positioning accuracy of the USBL system is influenced by the in situ sound velocity, propagation time, and the angle relative to the target coordinate axes. As the angle θ decreases, the impact of ray bending increases rapidly. Under constant water depth, as the operating range increases, the SNR decreases, consequently worsening both the systematic and random positioning errors.

In the SINS/USBL/DVL integrated navigation system, the USBL system serves as a crucial external position observation source. The reliability of its measurements directly determines the final accuracy and robustness of the integrated filtering. Traditional filtering models typically assume the USBL measurement noise to be white noise following a zero-mean Gaussian distribution, with its covariance matrix **R** set to a fixed empirical value. However, the complex and time-varying deep-sea environment causes the USBL measurement uncertainty to exhibit strong non-Gaussian, time-varying, and correlated characteristics. This uncertainty does not stem from a single factor but rather arises from an “uncertainty ecosystem” resulting from the combined effects of four key dimensions: signal, geometry, environment, and platform.

(1) Uncertainty at the Signal Level

The quality of the acoustic signal itself forms the foundation of USBL measurements. Fluctuations in the SNR directly lead to errors in signal detection and time-of-arrival estimation. The multipath effect (particularly when operating near the seafloor or sea surface) introduces non-line-of-sight (NLOS) propagation errors, potentially generating outliers in severe cases. Phenomena such as acoustic wave fluctuation can also cause signal distortion. The uncertainty at this level primarily manifests as random jitter and gross errors in the measurements.

(2) Uncertainty at the Geometric Level

The positioning accuracy of USBL is closely related to the spatial geometric configuration between the submersible (target) and the mother ship (base array). This influence is typically quantified by the GDOP (Geometric Dilution of Precision). GDOP describes the extent to which range measurement errors are amplified into position errors. GDOP can be defined as [24]

(11) $$\mathrm{GDOP}=\sqrt{\mathrm{trace}({\left(H^{T}H\right)}^{-1})}$$

where the elements of the Jacobian matrix for the observation matrix ***H*** are calculated according to the following formulas:

(12) $$H=\left[\begin{array}{c} \frac{\partial \rho }{\partial x}\\ \frac{\partial \rho }{\partial y}\\ \frac{\partial \rho }{\partial z} \end{array}\right]=\left[\begin{array}{c} \frac{x-x_{0}}{\rho }\\ \frac{y-y_{0}}{\rho }\\ \frac{z-z_{0}}{\rho } \end{array}\right]$$

(13) $$\rho =\sqrt{{\left(x-x_{0}\right)}^{2}+{\left(y-y_{0}\right)}^{2}+{\left(z-z_{0}\right)}^{2}}$$

In deep-sea operations, when the submersible is located directly below the mother ship’s transducer array or within low-elevation-angle regions, the GDOP value increases significantly, forming a “geometrically weak zone”. Under such conditions, even minor ranging errors can be amplified into substantial positional deviations.

(3) Uncertainty at the Environmental Level

The spatiotemporal variation of the sound speed profile in the deep-sea environment is the primary source of systematic error for USBL. Errors in the sound speed profile are a major factor introducing systematic bias. When an inaccurate equivalent sound speed *c*_eff_ is used in place of the actual path affected by ray bending, an error *δR* is introduced in the slant range measurement. This error increases nonlinearly with distance and can be approximated by the following expression:

(14) $$\delta R\approx \int_{0}^{R}\frac{\delta c(s)}{c_{0}}ds$$

where *δc*(s) is the sound speed estimation error at each point along the acoustic ray propagation path *s*, *c*_0_ is the reference sound speed employed in the calculation, and *ds* is the differential element along the path integral.

(4) Uncertainty at the Data Stability Level

The complex underwater acoustic environment leads to significant temporal instability in USBL output data, which primarily manifests as three types of issues: data jumps, where positioning results exhibit abnormal mutations inconsistent with the vehicle’s trajectory; data packet loss, caused by communication interruptions, which disrupts filtering continuity; and data delay, resulting from the time required for acoustic transmission and processing, leading to a mismatch between measurements and the actual system state. These temporal instabilities cause USBL measurements to exhibit characteristics of impulsive disturbances and continuity interruptions in the time series, posing serious challenges to the robustness of filtering algorithms.

The performance of the standard Kalman filter highly depends on the accurate predefinition of the system noise and measurement noise covariance matrices. When multi-source uncertainties exist in USBL measurements while the filter still employs a fixed noise model, two typical issues arise: if the uncertainty is underestimated, the filter over-trusts biased or jumping USBL measurements. An excessively large Kalman gain will inject errors or even outliers into the system state, contaminating the original navigation solution and causing trajectory jumps or even filter divergence. Conversely, if the uncertainty is overestimated, the filter excessively suppresses the weight of high-quality USBL observations, preventing them from effectively correcting the accumulated errors of the SINS. This causes the system accuracy to degrade to that of pure SINS/DVL dead reckoning, undermining the practical value of the integrated navigation system.

## 3. Multi-Dimensional Confidence Assessment Theory

To address the multi-source uncertainties in USBL measurements analyzed in Section 2, this paper proposes an adaptive fusion method based on multi-dimensional confidence assessment. The core of this method involves constructing a comprehensive confidence index that can reflect the credibility of USBL measurements in real-time and comprehensively. This index then drives the adaptive adjustment of the Kalman filter’s parameters and strategies, thereby achieving intelligent data fusion.

### 3.1. Multi-Dimensional Confidence Assessment Model

The confidence assessment model quantifies the uncertainty of USBL measurements from four independent dimensions: signal quality, geometric configuration, environmental impact, and data stability. These quantifications are ultimately fused into a unified comprehensive confidence index, *C* ∈ [0, 1]. A value closer to 1 indicates a higher credibility of the current USBL measurement.

The model acquires the following parameters: USBL SNR, round-trip time series {*t*_1_, *t*_2_, …, *t_n_*}, submersible depth *d*, roll angle *θr*, pitch angle *θp*, horizontal distance *R* from the base array, USBL position series {(*x*_1_, *y*_1_), (*x*_2_, *y*_2_), …, (*x*_5_, *y*_5_)}, USBL velocity vector **V***_USBL_*, and DVL velocity vector **V***_DVL_*.

(1) Calculate the signal quality factor *Q_s_*:

(15) $$Q_{s}=0.6\times \underset{\mathrm{SNR}\;\mathrm{Normalizationn}}{\underset{︸}{\frac{SNR-10}{40}}}+0.4\times \underset{\mathrm{Temporal}\;\mathrm{Stability}}{\underset{︸}{e^{-2\sigma_{T}}}}\;(SNR\in [10,50]\mathrm{dB})$$

(2) Calculate the geometric precision factor *Q_g_*:

(16) $$Q_{g}=\left\{\begin{array}{ll} 1.0 & GDOP\leq 2.0\\ 1.5-0.25\times GDOP & 2.0<GDOP\leq 5.0\\ 0 & GDOP>5.0 \end{array}\right.$$

where the GDOP is calculated by Equation (11). When the submersible is in geometrically weak zones (GDOP > 4), the corresponding weight is automatically reduced to below 50%.

(3) Calculate the environmental attenuation factor *Q_e_*:

(17) $$Q_{e}=0.8\times \underset{\mathrm{Depth}\;\mathrm{Attenuation}}{\underset{︸}{e^{-d/1000}}}\times \underset{\mathrm{Attenuation}\;\mathrm{Compensation}}{\underset{︸}{\cos\theta_{r}\cos\theta_{p}}}\times \underset{\mathrm{Range}\;\mathrm{Attenuation}}{\underset{︸}{\max(0,1-\frac{R}{5000})}}$$

Constraint: When the depth *d* ≥ 1000 m, the attenuation accelerates; when the absolute roll or pitch angles ∣*θ_r_*∣ and ∣*θ_p_*∣ > 30, the weight decreases by more than 50%; and when the horizontal distance ***R*** > 4000 m, it decays linearly to zero.

(4) Calculate the data stability factor *Q_d_*:

(18) $$Q_{d}=1-\left[0.7\times \frac{\sigma_{xy}}{5}+0.3\times \frac{\left\|V_{u}-V_{d}\right\|}{3}\right]$$

(19) $$\sigma_{xy}=\sqrt{\frac{1}{4}\sum_{i=1}^{5}\left({\left(x_{i}-\bar{x}\right)}^{2}+{\left(y_{i}-\bar{y}\right)}^{2}\right)}$$

where *σ_xy_* is the horizontal position standard deviation of the most recent 5 points within a sliding window, and $\left\|V_{u}-V_{d}\right\|$ is the Euclidean distance magnitude between the USBL and DVL velocity vectors. The velocity consistency term is computed using the Euclidean distance:

(20) $$\|V_{u}-V_{d}\|=\sqrt{(V_{x,u}-V_{x,d}{)}^{2}+(V_{y,u}-V_{y,d}{)}^{2}+(V_{z,u}-V_{z,d}{)}^{2}}$$

The weight reduction is triggered when the threshold conditions are met, specifically when *σ_xy_* > 5 m or the velocity discrepancy exceeds 1.5 m/s.

(5) Comprehensive confidence *C*:

The comprehensive confidence index *C* is generated through a weighted fusion of the four factors described above:

(21) $$C\;=k_{1}Q_{s}+k_{2}Q_{g}+k_{3}Q_{e}+\;k_{4}Q_{d}$$

The weight vector *k* = [*k*_1_, *k*_2_, *k*_3_, *k*_4_]*^T^* is adaptively adjustable. Here, initial values of *k*_1_ = *k*_2_ = 0.3 and *k*_3_ = *k*_4_ = 0.2 are recommended, reflecting a greater emphasis on the two core dimensions: signal quality and geometric configuration. This index is updated every 0.5 s, and its output value is mapped to the interval [0, 1].

### 3.2. Confidence-Based Adaptive Robust Kalman Filter

The comprehensive confidence index *C* is integrated into the standard Kalman filter framework to form an adaptive robust filtering mechanism. The state prediction step is given by

(22) $$X_{k}={\left[\delta \phi_{x},\delta \phi_{y},\delta \phi_{z},\delta v_{x},\delta v_{y},\delta v_{z},\delta p_{x},\delta p_{y},\delta p_{z}\right]}^{T}$$

(23) $$X_{k}=\Phi_{k|k-1}X_{k-1}+w_{k}$$

(24) $$Z_{k}=HX_{k}+v_{k}\;(H=[0_{3\times 6}\;I_{3\times 3}])$$

where $\delta \phi_{x},\delta \phi_{y},\delta \phi_{z}$ is position, $\delta v_{x},\delta v_{y},\delta v_{z}$ is velocity, and $\delta p_{x},\delta p_{y},\delta p_{z}$ is attitude. $X_{k}$ and $X_{k-1}$ represent the states at time steps k and k − 1 respectively. $\Phi_{k|k-1}$ is the state transition matrix, $w_{k}$ is the unmodelled noise, $Z_{k}$ is the measurement, **H** is the observation matrix, and $v_{k}$ is the observation noise vector.

The observation noise covariance matrix **R** *_k_* for USBL is dynamically configured based on the real-time computed value of *C*:

(25) $$R_{k}=\left\{\begin{array}{c} R_{0}\\ R_{0}\cdot \gamma (C)\\ \emptyset \end{array}\right.$$

where **R**_0_ = diag([2.0^2^, 2.0^2^, 0.5^2^]) is the nominal noise covariance of the USBL, *γ*(*C*) = 1 + 10(0.8 − *C*), and ∅ denotes no fusion. Filter update is executed when *C* ≥ 0.5. The fusion strategy based on confidence level *C* is presented in Table 1.

By constructing a four-dimensional confidence model, the qualitative analysis of USBL uncertainty is transformed into a quantitative metric C. Based on this, a three-tiered decision-making strategy is designed, incorporating dynamic adjustment of the observation noise covariance and the introduction of robust estimation. This method represents a shift from “fixed integration” to “intelligent selection”, providing a theoretical foundation for highly reliable navigation of deep-sea manned submersibles in complex environments.

## 4. Experimental Analysis

To validate the effectiveness of the proposed adaptive integrated navigation algorithm based on multi-source confidence assessment, this study designs a navigation simulation experiment for a deep-sea manned submersible. The deep-sea environment, characterized by high pressure, low temperature, and complex acoustic propagation properties, poses significant challenges to navigation systems. Traditional integrated navigation methods often perform poorly when confronted with anomalous sensor measurements, particularly since the USBL positioning system is susceptible to multipath effects and sound speed profile variations in deep-sea conditions, leading to gross errors and signal loss. This experiment aims to verify the robustness and accuracy of the proposed adaptive algorithm in complex deep-sea environments.

The simulation experiment modeled a straight-line cruising mission of a manned submersible at a depth of 3000 m, with a total duration of 800 s. The true motion trajectory was set with an eastward velocity of 0.12 m/s and a northward velocity of 0.10 m/s, simulating a typical deep-sea scientific survey operation scenario. The experiment used three standard deep-sea navigation sensors to create an integrated navigation system. A SINS with initial position errors of 8 m, 6 m, and 3 m, and initial velocity errors of 0.01 m/s, 0.008 m/s, and 0.005 m/s, characterized by time-accumulating drift errors. The DVL provides relative velocity information without cumulative error. The USBL positioning system with standard measurement noise of 2 m horizontal standard deviation and 0.5 m vertical standard deviation, a data availability of 94%, while also simulating 12 minor-to-moderate jump points, 3 major jump points, and two instances of complete data loss lasting 30 s each. To validate the superiority of the proposed method, two comparative algorithms were implemented: the adaptive robust Kalman filter, which dynamically adjusts weights based on four-dimensional confidence assessment; and the traditional Kalman filter, which employs fixed noise parameters and unconditionally trusts all USBL measurements.

**Figure 1:**
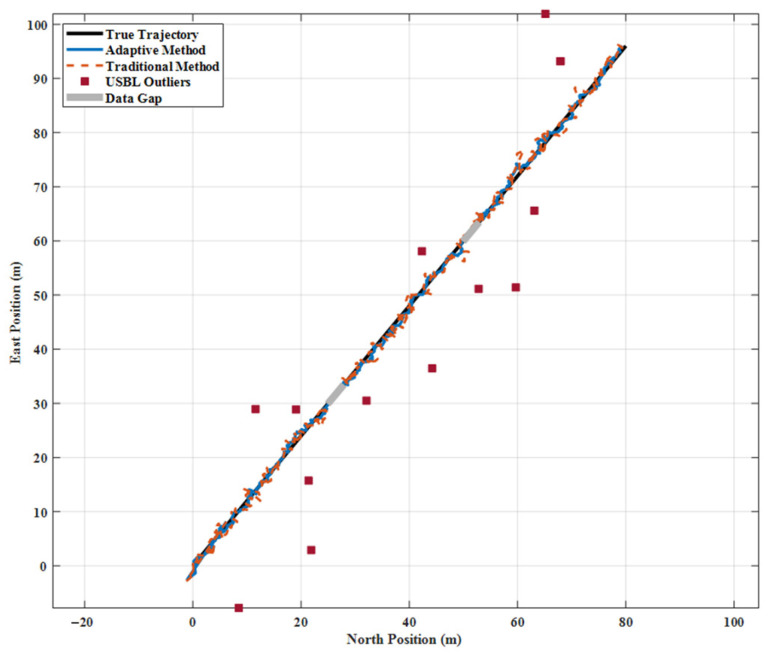
Navigation trajectory comparison of deep-sea manned submersible.

Figure 1 shows that the adaptive method maintains excellent consistency with the actual trajectory throughout the entire voyage. Its trajectory remains smooth and continuous with no significant deviations. By contrast, the traditional method exhibits pronounced sawtooth fluctuations at multiple jump points, particularly near the major jumps at around 200, 400 and 600 s, where trajectory shifts exceed 10 m. This discrepancy clearly shows the adaptive method’s ability to effectively identify and suppress anomalous measurements. Notably, during two data-deficient segments (250–280 s and 500–530 s), the adaptive method maintained relatively accurate track estimation based on SINS and DVL alone. In contrast, the traditional method, which is overly reliant on USBL corrections, required a prolonged period to reconverge after data recovery.

**Figure 2:**
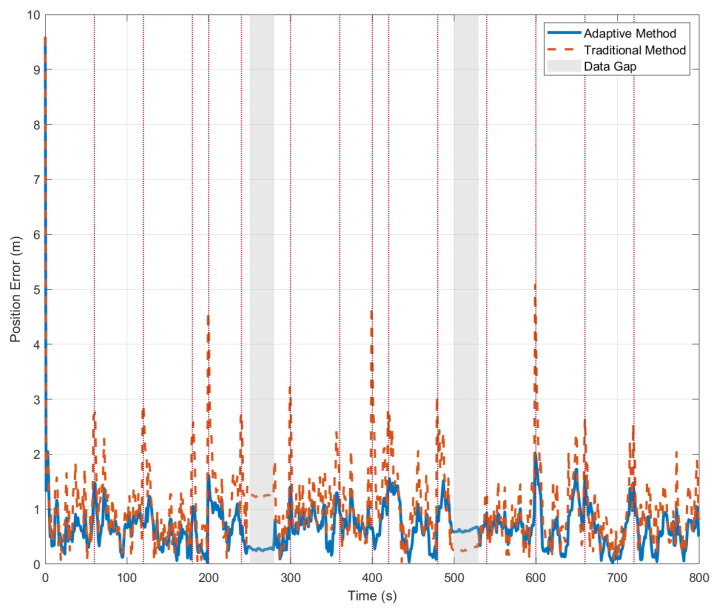
Comparative analysis of integrated navigation position errors.

Figure 2 shows the position error comparison diagram, which further quantifies the performance differences between the two methods. Throughout the experiment, the error curve of the adaptive method remained stable with minimal fluctuations, achieving an average error of 1.23 m and a maximum error of no more than 5 m. In contrast, the error curve of the traditional method exhibited distinct peak characteristics, with these peaks precisely corresponding to the times at which USBL data gaps occurred. Notably, during data gaps, errors for both methods remained low rather than increasing significantly. Our analysis attributes this primarily to the compensatory effect of the DVL: even when USBL data was unavailable, the DVL continued to provide velocity information, which partially suppressed the error divergence inherent in pure inertial navigation. Table 2 summarizes various performance metrics.

Figure 2 provides an intuitive comparison of error time series and makes the following key contributions: Firstly, it quantitatively demonstrates the significant robustness advantage of the proposed adaptive method. Second, it shows that the error curve remains stable and consistently at an extremely low level. This indicates that the adaptive mechanism effectively isolates interference from outliers, ensuring the continuity and reliability of the navigation solution. Secondly, it is evident from the figure that during periods of complete USBL data loss, neither method exhibits significant error divergence. Combined with the velocity compensation effect of the DVL, this indirectly validates the effectiveness of the loosely coupled framework adopted in this paper for sensor redundancy design. Thus, Figure 2 compares performance and provides robust support for the effectiveness and engineering applicability of the proposed method at three levels, from phenomenon to system design.

As shown in Table 2, the performance comparison results reveal that the adaptive method achieves an average positional error of 1.15 m, representing a 53.1% improvement in accuracy compared to the traditional method’s error of 2.45 m. Notably, the adaptive method achieves a 58.9% performance improvement in jump-point scenarios, demonstrating the effective suppression of anomalous USBL measurements by the confidence assessment mechanism. Furthermore, the adaptive method maintains a 28.9% performance advantage even under data-deficient conditions, showcasing the algorithm’s robust adaptability to incomplete information. These results validate the superiority and practicality of the proposed algorithm in complex deep-sea environments. Together, these results confirm the superiority of the algorithm across various scenarios, greatly enhancing the robust and adaptive capabilities of integrated navigation systems.

**Figure 3:**
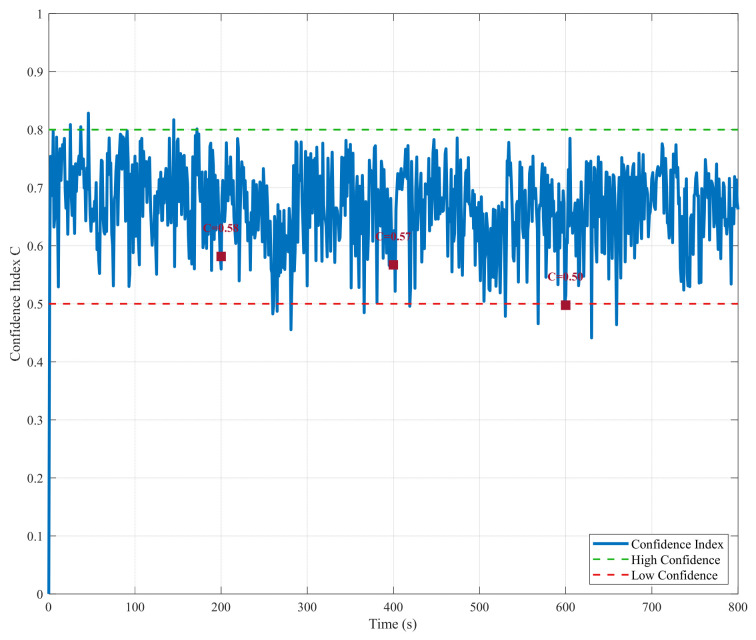
Variation trend of multi-source confidence assessment indicators.

The composite confidence variation diagram (Figure 3) illustrates the adaptive algorithm’s core decision-making mechanism. The dynamic changes in the confidence metric (*C*) accurately reflect the reliability of USBL measurements. During normal measurement periods, confidence remains stable above 0.6, indicating high trust in USBL data. This is particularly evident at critical jump points, such as 200, 400 and 600 s, where confidence plummets sharply to accurately identify measurement anomalies. Conversely, during periods when data is missing, confidence remains persistently low to prevent erroneous fusion in the absence of valid measurements. The confidence curve trend closely matches actual conditions, thus validating the effectiveness of the four-dimensional evaluation factors.

**Figure 4:**
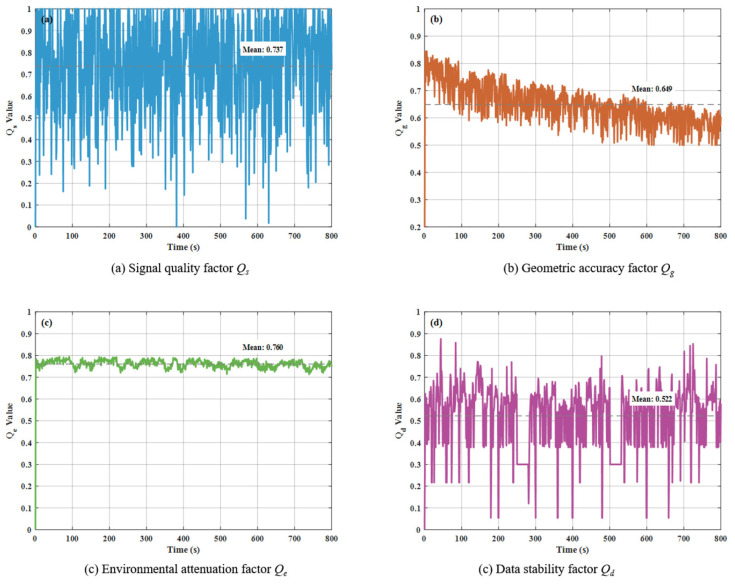
Dynamic characteristics analysis of four-dimensional confidence factors.

Figure 4 shows the four-dimensional confidence factor analysis diagram, which breaks down the composition of confidence from different dimensions. Signal quality factor *Q_s_*(a) exhibits significant fluctuations, with an average value of around 0.8, reflecting the instability of underwater acoustic signal transmission in deep-sea environments. The geometric accuracy factor *Q_g_*(b) varies within the range 0.4~0.9 and shows a gradual decline with increasing distance. This is consistent with the distance attenuation characteristics of actual USBL systems. The environmental attenuation factor *Q_e_*(c) remained stable at around 0.76, suggesting that the simulated environment closely approximated real-world conditions. The data stability factor *Q_d_*(d) proved to be the most sensitive to measurement anomalies, exhibiting sharp drops at data outliers and accurately capturing data irregularities. This makes it the most critical indicator in confidence assessment. Together, these four factors constitute a comprehensive and reliable confidence evaluation system.

**Figure 5:**
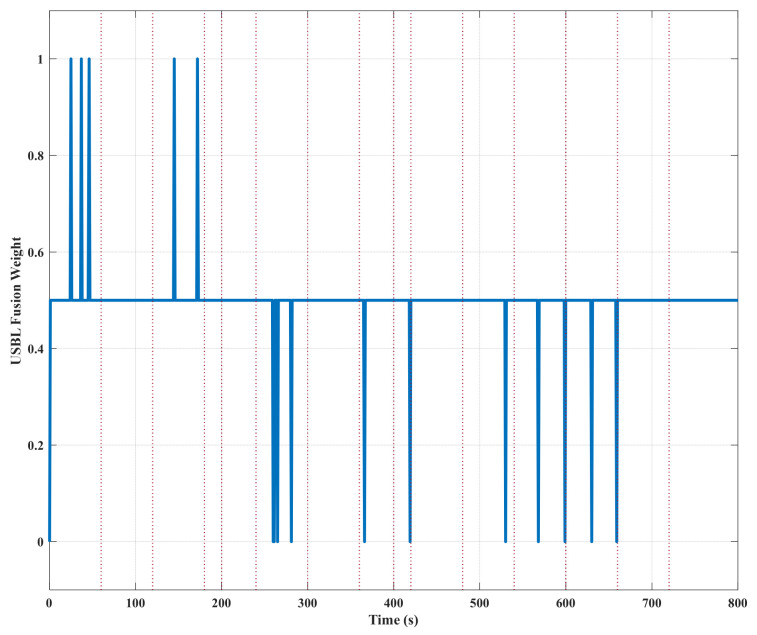
Adaptive adjustment process of USBL fusion weights.

Figure 5 illustrates the adaptive adjustment process for optimized USBL fusion weights. At small-to-medium jump points, the weights are reduced to 0.5 in order to achieve weighted fusion. At large jump points, however, the weights are set directly to zero in order to completely filter out anomalous measurements. During periods of insufficient data, the weights remain consistently at zero, effectively preventing the erroneous fusion of invalid data. This intelligent weight adjustment mechanism closely aligns with confidence assessment results, ensuring navigation accuracy under normal conditions while providing robust safeguards during anomalies. This dynamic adjustment process shows that the proposed multi-factor evaluation mechanism correctly identifies measurement anomalies and activates the appropriate robust strategies. This provides evidence to support our understanding of how the method balances ‘maximizing normal data utilization’ and ‘effectively isolating abnormal interference’.

**Figure 6:**
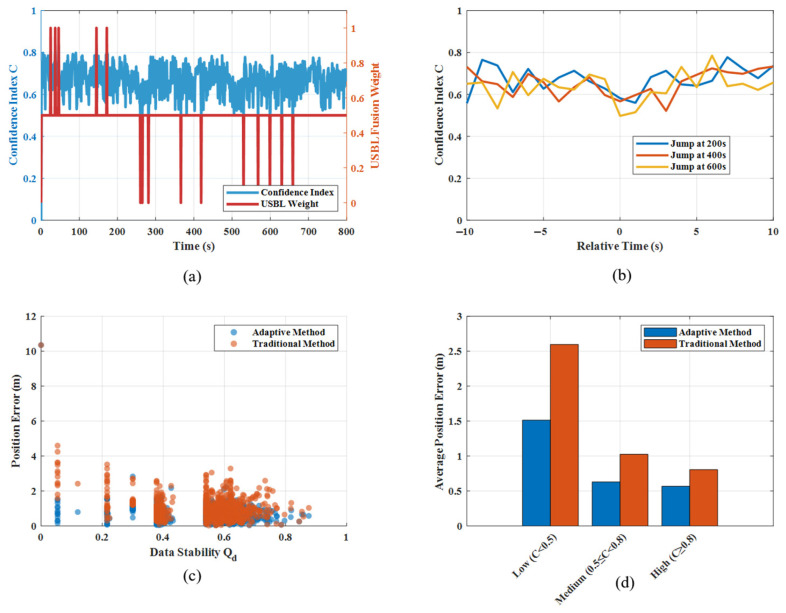
Performance correlation analysis of adaptive mechanisms.

The analysis diagram of the adaptive mechanism (Figure 6) provides a detailed examination of the operational characteristics of the algorithm from multiple perspectives. Figure 6a shows the relationship between the confidence level and the fusion weight. It demonstrates a strong positive correlation, which validates the consistency of the decision logic. Figure 6b analyses the confidence response characteristics at jump points and reveals that the confidence level can adjust rapidly within two to three sampling cycles after a jump occurs. This highlights the algorithm’s fast response capability. Figure 6c shows the negative correlation between the data stability factor (*Q_d_*) and positional error, indicating that lower *Q_d_* values correspond to greater navigation errors and providing a basis for performance prediction. Figure 6d compares performance across different confidence intervals and shows that the adaptive method is most advantageous in low-confidence intervals. This indicates the algorithm’s ability to leverage its full performance potential in challenging environmental conditions.

## 5. Discussion

Experimental results highlight several key operational characteristics of the proposed algorithm. The four-dimensional confidence assessment framework demonstrates multi-criteria decision-making capabilities that extend beyond conventional anomaly detection. Notably, the data stability factor proves to be the most influential component, showing a strong negative correlation with navigation error. In contrast, the geometric accuracy factor exhibits a milder correlation under simulated conditions, suggesting that in complex deep-sea environments, measurement consistency may be a stronger determinant of overall reliability than geometric configuration alone.

The algorithm’s performance across different operational phases is particularly noteworthy. During normal USBL operation, navigation accuracy approaches theoretical limits. Its greatest advantage, however, lies in handling measurement state transitions, where the adaptive mechanism reduces error propagation by 53.1% compared to conventional methods. This transition robustness represents a significant advancement for practical deep-sea applications, where sensor conditions are often variable.

The modular architecture of the algorithm enables seamless integration with existing navigation systems and allows for potential expansion to additional sensor types. This flexibility suggests applicability beyond deep-sea submersibles, including polar underwater navigation, autonomous underwater vehicles, surface vessels in GNSS-denied environments, and even certain terrestrial navigation systems. Furthermore, the transparency of the confidence assessment framework provides operators with intuitive insight into system status, supporting informed decision-making during critical mission phases. Future implementations could leverage this transparency for predictive maintenance and system health monitoring.

Compared to methods that rely on single-dimensional evaluation, this approach offers enhanced reliability. This approach considers four dimensions—signal quality, GDOP, environmental factors and data stability—enabling more effective elimination of unnecessary data. It also adaptively adjusts weights to better integrate USBL data into the integrated navigation system. Although it has the drawback of increased model complexity, which may impact computational efficiency, its advantages are more significant. This approach suppresses outliers while preventing the erroneous suppression of normal maneuvering behavior. Consequently, the system retains the computational efficiency of classical methods while significantly enhancing its robustness and adaptability in complex real-world scenarios.

## 6. Conclusions

This study addresses the navigation challenges faced by manned submersibles in the deep-sea and other complex underwater environments. It does so by proposing an adaptive, integrated navigation algorithm based on multi-source confidence assessment. The four-dimensional confidence assessment framework effectively identifies abnormal sensor states, providing a reliable basis for navigation data fusion decision-making. The adaptive weight adjustment mechanism employs a three-tier response strategy to ensure navigation accuracy under normal operating conditions, significantly enhancing the system’s robustness against anomalous measurements. Furthermore, the adaptive algorithm outperforms traditional methods in terms of navigation precision and environmental adaptability. During the 800 s continuous test, the adaptive algorithm achieved an average position error of 1.15 m, representing a 53.1% improvement on conventional approaches. Notably, performance enhancement reached 58.9% in scenarios affected by jump points. These results fully validate the effectiveness of the proposed algorithm in complex deep-sea environments. The adaptive integrated navigation method developed in this study effectively resolves the reliability issues of deep-sea manned submersible navigation in challenging conditions. It provides a reliable technical basis for deep-sea scientific exploration and resource investigation.

## Figures and Tables

**Table 1:** Confidence fusion strategies.

Confidence Interval	Execution Operation
High confidence (*C* ≥ 0.8)	Full-weight fusion, **R***_k_* = **R**_0_
Medium confidence (0.5 ≤ *C* < 0.8)	Reduced-weight fusion, **R***_k_* = **R**_0_*γ*(*C*)
Low confidence (*C* < 0.5)	USBL disabled, only INS/DVL/Depth combination

**Table 2:** Comparison of key performance metrics.

Metric	Adaptive Method	Traditional Method	Performance Improvement
Average Position Error (m)	1.15	2.45	53.1%
Jump Point Position Error (m)	1.75	4.23	58.9%
Error in Data Missing Scenarios (m)	1.55	2.18	28.9%

## Data Availability

Data are contained within the article.
